# The threat to person-centered care: how censoring healthcare language endangers public health

**DOI:** 10.1093/pubmed/fdaf061

**Published:** 2025-11-18

**Authors:** Serena Barello, Mateus Eduardo Romão

**Affiliations:** Department of Brain and Behavioural Sciences, University of Pavia, Pavia, 27100, Italy; Department of Brain and Behavioural Sciences, University of Pavia, Pavia, 27100, Italy

**Keywords:** public health, health services, ethics

In early 2025, a sweeping executive order from the Trump administration mandated the removal of so-called “woke” terminology from federal agencies.^1^ This directive targeted words and phrases related to diversity, equity, and inclusion, erasing terms central to modern healthcare—*person-centered care* (PCC), *health equity*, *advocacy*, and *inclusivity.* Overnight, key concepts that had shaped medical education, public health policy, and clinical practice disappeared from official guidelines, websites, and research funding frameworks.

This is more than a bureaucratic adjustment; it is a fundamental shift in healthcare philosophy. The elimination of these terms alters how health is discussed, measured, and addressed at an institutional level. The consequences will be felt in how care is delivered, how researchers study health disparities, and how public health interventions are implemented. Most critically, erasing PCC threatens to reverse decades of progress in making medicine more responsive, ethical, and humane.

PCC is not a political slogan but a well-established, evidence-based model that places patients at the heart of medical decision-making. It ensures healthcare adapts to individuals rather than imposing one-size-fits-all protocols and acknowledges that values, preferences, and social circumstances must be integrated into clinical decisions.^2^ Removing PCC from official discourse weakens the patient engagement movement and reinstates a more paternalistic, provider-driven model of care.

Without PCC in health policies, the balance between medical expertise and patient autonomy is at risk. Clinicians and patient advocacy organizations who rely on institutional support to advocate for patient-centered approaches may face pressure to prioritize efficiency, cost-cutting, or standardized treatment models that fail to address individual needs. This shift could weaken shared decision-making, reduce patient adherence to treatment plans, and widen health disparities—especially for vulnerable populations already struggling to access tailored care. From a workforce perspective, these changes may contribute to job dissatisfaction, poorer performance, and burnout.^3^

The consequences are particularly dire for chronic disease management. Conditions like diabetes, hypertension, and cancer require sustained collaboration and alliance between patients and providers. PCC ensures treatment plans consider financial barriers, lifestyle constraints, and cultural factors influencing adherence. Ignoring these factors leads to rigid, ineffective care. Research consistently shows that when patients feel unheard and disempowered, they are less likely to follow treatment recommendations, resulting in worse outcomes and increased healthcare costs.^4^

Beyond individual patient care, removing PCC-related language undermines public health efforts to address systemic inequities. Many health equity programs recognize that medical care alone is insufficient for good health—factors like housing, food security, and education play crucial roles. Without language to describe these influences, public health officials will struggle to justify interventions targeting the root causes of health disparities. If policymakers can no longer reference social determinants of health, how can they advocate for policies addressing poverty-related illness? If they are forbidden from discussing health equity, how do they argue for expanding access to care for underserved communities?

This intentional erasure undermines the moral imperative of public health, which is essentially committed to advancing and protecting health among all people, particularly those most disadvantaged. When policymakers erase conversations about equity or inclusion, they undermine the ethical obligation of public health professional practice: to provide equity, justice, and dignity to healthcare access.

This omission is not incidental—it reflects a deliberate policy shift with real-world consequences. The absence of references to historically underserved populations from federal resources does not alter the reality that these groups experience higher rates of chronic illness, mental health challenges, and barriers to care. Suppressing data on racial and socioeconomic health disparities does not eliminate inequities—it only makes them more difficult to identify, address, and rectify.

The impact will also be felt in medical education. Future doctors, nurses, and public health professionals are shaped by the language and frameworks they are taught. If PCC and health equity disappear from federally funded training programs, new clinicians may enter the workforce with little understanding of how to engage with diverse patient populations. If this trend continues, future healthcare providers will be less prepared to meet the needs of the communities they serve, limiting their ability to effectively engage patients.

Clarifying what these frameworks represent in educational curricula diminishes the competence, capacity, and resiliency of future public health experts. Suppose training is not based on ethical principles of inclusivity, equity, and patient choice. In that case, healthcare workers are unlikely to have the necessary skills and resources to effectively address disparities in health and respond to ethically challenging clinical situations.

The ethical consequences of this shift cannot be overstated. PCC is not just a strategy for better health outcomes—it is a fundamental principle of medical ethics. Respect for patient autonomy, the right to informed consent, and the duty to provide non-discriminatory care are not optional values; they are the foundation of modern medicine. Erasing the language of PCC undermines these ethical commitments, risking a future in which patients become passive recipients of care rather than active participants in their health.

The *Quintuple Aim for Health Care Improvement* provides a crucial framework for resisting this regression. Expanding upon the Triple Aim and Quadruple Aim, this model emphasizes health equity as a fundamental component of effective healthcare.^5^ By integrating equity into the core goals of improving population health, enhancing patient experience, reducing costs, and supporting provider well-being, the Quintuple Aim underscores the need for inclusive, patient-centered language and policies. Without this focus, disparities will deepen, and the healthcare system will fail its most vulnerable patients.

Healthcare professionals, educators, and policymakers must actively resist the erosion of PCC and health equity. The fight for PCC is not just about language—it is about the well-being of millions who depend on a fair, responsive, and compassionate healthcare system. To ensure an ethical and effective future for medicine, we must protect and uphold the principles that prioritize participatory care, patient needs, and equitable care.

## Footnotes

The opinions here are solely those of the authors and do not represent any organization with which they are affiliated.

## Notes

**Serena Barello**, Associate Professor of Health Psychology, Department of Brain and Behavioral Sciences, University of Pavia.

**Mateus Eduardo Romão**, PhD Candidate in Psychology, Department of Brain and Behavioral Sciences, University of Pavia.
